# Young Children’s Nutrition During the COVID-19 Pandemic Lockdown: A Comparative Study

**DOI:** 10.1007/s10643-021-01192-3

**Published:** 2021-04-28

**Authors:** Raja Omar Bahatheg

**Affiliations:** King Saud University, Riyadh, Saudi Arabia

**Keywords:** Nutrition, Children, COVID-19, Lockdown, Saudi Arabia

## Abstract

During the COVID-19 pandemic, most countries took precautionary steps to save their citizens by initiating a lockdown and stopping all social activities by closing schools, companies, entertainment places, markets, gardens, and other social gathering places. As children stayed at home with no physical activities, their weight may have increased. The purpose of this study was to examine the link between fast food, sugars, or soft drinks and the ongoing domestic lockdown of the COVID-19 pandemic. This phenomenon was studied in three different cities from three different countries (Saudi Arabia, Britain, and Turkey) from the perspective of children’s parents. The study sought to address three research questions regarding children’s well-being during the COVID-19 lockdown period. First, was children’s nutrition affected during this period? Second, did children's weight increase? Third, were there any statistically significant differences in children’s dietary patterns based on their gender and nationality? A questionnaire was administered to 330 parents of children aged four to seven years in the three targeted countries. The study found that most parents cared about their children's nutrition and prepared food at home (96.1%) during the lockdown. Sixty-three percent of parents indicated that children did not gain weight. Additionally, differences in children’s nutritional systems were found between Saudi and Turkish children; the nutritional system of the Turkish children was better than that of Saudi children during the lockdown. Additionally, there were statistically significant differences in children’s nutrition due to gender, with better nutrition for boys than for girls.

## Introduction

On March 11, 2020, the World Health Organization announced that the novel COVID-19 virus had become a global pandemic. The rapid spread of the virus has resulted in some urgent temporary measures such as lockdowns in schools and universities all over the world. Most countries locked down educational institutions until further notice, with the provision of appropriate alternatives, such as the activation of “virtual schools” and distance learning classes.

Children and adolescents' lifestyle behaviors, such as their physical activity and nutrition, may have been drastically impacted by prolonged school closures and home confinement during the COVID-19 pandemic. During the pandemic, the concept of self-care has grown. According to the National Alliance of Mental Health, the concept of self-care is a decision you take to improve your health, and it should be practiced daily. It includes body movement, health, nutrition, sleep, rest, and physical touch. The family or the children’s caregivers should educate children about health care during this period, especially eating behaviors. These behaviors include eating healthy food and adhering to healthy habits such as personal hygiene. Moreover, the partnership between UNICEF and the World Food Program concentrated on intensive programs to protect, promote, and support optimal nutrition practices recommended for infants and young children, while adhering to health precautions, including stay-at-home procedures (UNICEF, 2020). Most governments all over the world imposed a lockdown during the COVID-19 pandemic, which has resulted in widespread disruption of normal daily life. In India, for example, Upadhyay et al. (2020) stated that the virus spread led to the unavailability of food and a lack of nutrition-related services, which, in turn, affected the overall nutritional status of children across India. These studies discussed the impact of the pandemic on the child's integrated growth plan and on the midday meal services scheme, which resulted in the risk of an inadequate child nutrition system.

In addition, with the spread of COVID-19, children are obliged to stay home for a long period of time because of the lockdown. Social media and magazines in Saudi Arabia focused on how the lockdown was causing people to gain weight (Middle East Newspaper, 2020; Al- Majalah Alttebiah 2020). Social media refers to websites, apps, and electronic tools that allow for digital social interaction. Today, most people use social media to access news and health information (Anderson & Jiang, 2018). The author’s observation of social media such as Twitter—Snapchat, WhatsApp, and Facebook, indicated that most families were discussing food preparation, which they believed was associated with the weight gain of most family members. Additionally, Rundle et al. (2020) anticipated that the COVID-19 pandemic will likely double out-of-school time this year for many children and will exacerbate the risk factors for gaining weight that are associated with summer holidays. This raises the question of how children’s nutrition and weight have been affected during the COVID-19 pandemic stay-at-home lockdown period. Limited data are available that connect the current COVID-19 pandemic with weight gain and the alteration of children’s nutrition.

Rundle et al. (2020) argued that, due to social distancing orders put in place during the COVID-19 lockdown, many children experienced higher-calorie diets during the pandemic response. In addition, news media outlets reported that the general public panicked by purchasing necessities and emptying supermarket shelves to stockpile goods, as seen with a 7.5% increase in grocery spending through March 27, relative to the previous year (Baker et al. 2020). Baker et al. (2020) also stated that during the lockdown, households were told to prepare their food at home and stay inside their homes for many weeks while only going outside for necessities such as grocery shopping, hospital visits, etc. Hence, poor eating habits may be formed and stabilized in childhood and adolescence. If there is no intervention to correct it, dietary habits can become a source of health problems in the future (Naserpoor et al. 2018; Pirzadeh et al. 2011). This research intends to fill the gap in the current literature by examining if there is an association between young children in some countries gaining weight due to an increase in consumption of high-calorie food prepared at home during the lockdown period of COVID-19.

Furthermore, children are social distancing and their eating habits may change during the COVID-19 lockdown. Additionally, there is an increase in the number of overweight individuals in some industrialized countries (Milano Cities Changing Diabetes Atlas 2020). Staying home for a long period of time might affect children’s weight. Young children are unable to choose the food they eat, just as they are unable to predict the long-term effects of their nutritional behavior; therefore, children need support and education in this regard (Romanos-Nanclares et al. 2018). Gaining weight during childhood has a negative impact on a child's health, as it may lead to negative effects such as spinal deformities, diabetes, cardiovascular diseases, and lung diseases that cause the child to develop asthma (Gondek et al. 2018).

COVID-19 lockdown changed people’s lifestyles, potentially affecting the health, safety, and daily lives of all individuals. This research aims to discover if children’s nutritional lifestyles changed during COVID-19. Do children eat more unhealthy foods with high calories or continue their healthy lifestyle? Do children who stay home during COVID-19 gain weight? Children tend to experience unhealthy weight gain not during the school year, but rather primarily during the summer months when they are out of school (Rundle et al. 2020). One of the basic strategies to prevent obesity is to balance meals with moderate calories (Kupczak-Wiśniowska et al. 2017). The main risk factor for obesity is an unhealthy lifestyle (e.g., eating extra calories, inactivity, and unhealthy nutrition). A healthy lifestyle plays a vital role in improving life expectancy, and is associated with reduced risk of death and recurrence of many diseases (Armoon & Karimy, 2019).

### Problem Statement

The Food and Agriculture Organization (FAO) of the United Nations argued that when schools are closed or suspended, parents, caregivers, and children should be educated and informed about the importance of having safe, healthy meals. In addition, the FAO highlighted the importance of personal hygiene and physical exercise for school-age children. During the COVID-19 pandemic, some researchers conducted studies that addressed the issue of children who live in poor regions, and the extent of their vulnerability to this pandemic. On the other hand, few studies dealt with the health habits of children during the stay-at-home period. Research has also been conducted to link staying at home and gaining weight, obesity, and eating unhealthy food (Alghadir et al. 2016; Finucane et al. 2011; Epstein et al. 2008; Jackson 2009; Marshall et al. 2004). In addition, researchers found that eating meals while engaging in technologies such as videos, DVD players, videogames, and television, were prime factors for increased risks of developing obesity (Alghadir et al. 2016; Lissner et al. 2012). Therefore, it is crucial to examine the changes in children's health and eating habits during the stay-at home period, and highlight the influence of the lockdown on young children in different areas of the world. Three purposeful samples were drawn from different cultures: one of them is the Saudi culture (Riyadh city) from the Middle East, the second is the British culture (London city) as a sample from Western culture; and third is the Turkish culture (Istanbul city) since it contains Eastern and Western cultures in one place, and is in the middle between West and East. In addition, research in Saudi society found that when combined, a higher duration of television viewing and consumption of high-fat fast foods and high-sugar drinks, posed a major threat to the younger generation (Alghadir et al. 2016). Conversely, research on Turkish students and their eating habits and body weight status, found that about 70% of the students (69.9%) were of normal weight and more than one-fourth (25.7%) were underweight. In addition, the UK has one of the highest levels of childhood obesity in Europe (National Obesity Forum 2015).

### Objectives

Obesity research studies are associated with children consuming fast food and sweetened juices or drinks. The ongoing stay-at-home procedures made children have home-prepared food, prompting the research questions in the current study. Families may be offering children healthy homemade food or fast food prepared at home. Additionally, the question remains whether foods prepared at home will lead to children being overweight, or whether it may help in establishing good eating habits through healthy meals offered at home. The current study aimed to investigate children’s nutrition based on parents’ background culture during the COVID-19 lockdown.

### Questions

The current study aimed to investigate changes in children’s nutritional systems during the COVID-19 pandemic stay-at-home period. The research questions were as follows:

- Has children’s nutrition been affected during the COVID-19 pandemic stay-at-home period in Saudi Arabia, the United Kingdom, and Turkey?
- Are there any statistically significant differences in children’s nutrition that can be attributed to nationality during the COVID-19 pandemic stay-at-home period in Saudi Arabia, the United Kingdom, and Turkey?
- Are there any statistically significant differences in children’s nutrition that can be attributed to the gender variable during the COVID-19 pandemic lockdown in Saudi Arabia, the United Kingdom, and Turkey?

## Materials and Methods

The current research is an exploratory survey designed to investigate the health of Saudi, British, and Turkish children (four to seven years old) during the COVID-19 lockdown from the perspectives of their parents. This study was approved by the local ethical review board and authorized by the Ministry of Education. In addition, to obtain parental permission, it was advised that a parent should read and go through the survey beforehand, and once they approved their child’s participation, they read it to their child and recorded their answers. All subjects included in this research provided written informed consent before participation, and all data were used for this research and kept confidential. The principal investigator sent the IRB approval with the survey to teachers and academic members in the UK and Turkey. The survey was distributed via Google Forms (because of the restrictions due to the pandemic) to different schools along with families and friends, targeting boys and girls aged four to seven years.

The survey used was designed and reviewed by a panel of experts, comprised of a jury of faculty members at the Education Faculty of [blinded for review]. A survey was prepared as it was deemed the most appropriate means of collecting the data necessary to prove or reject the study hypotheses. The survey questions were designed according to the study goals and hypotheses, and based on cumulative knowledge obtained from theoretical readings within the framework of the subject of the study that contained a set of phrases and response choices. The respondents were asked to answer according to what they deemed appropriate for themselves and applied to their reality.

The survey was sent to a sample of Saudi, Turkish, and British families through a Google Form randomly, containing a series of yes and no questions. To ensure the accuracy of the survey phrases in measurement variables, the validity of the tool was verified by a group of specialized faculty members and specialists in the College of Education, Department of Early Childhood at King Saud University. Some adjustments were made to the tool, in formulating some vocabulary, removing other vocabulary, and adding new vocabulary. After confirming the validity of the scale, Cronbach's alpha was calculated as a measure of the stability of the respondents' answers. The internal consistency of the study sample was 0.82, which indicates a high degree of consistency and can be relied upon in the field based on the reliability of its results. Internal reliability, which measures the degree of correlation between different items of the questionnaire, was assessed with a Pearson's correlation coefficient for knowledge scores, and a statistical significance level (0.01). Validity was assessed by Cronbach's alpha (0.65), which indicates that the study instrument has acceptable stability in the field application of the study.

### Participants

The randomly-chosen research sample consisted of parents of 330 children from the studied countries as follows: Saudi Arabia (198), British (62), and Turkish (70). Most of the study sample was comprised of Saudi children (60.0%), while the percentage of Turkish children was 21.2%, and the percentage of British children was 18.8%. Mothers comprised the majority of the study sample: there were 313 mothers (94.8%) and 17 fathers (18.8%). The majority of the children were seven years old (122 children, 37.0%), with four-year-olds accounting for 26.4% (87 children), five-year-olds for 19.7% (65 children), and six-year-olds for 17.0% (56 children). The total number of male children participating in the study was 160 (48.5%), whereas the total number of female children participating was 170 (51.5%).

### Data Analysis

Data collected were analyzed using SPSS software. Pearson’s correlation was used to assess the external construct validity, in which a statistical function belongs at the significance level (0.01) of all the axes of the items. In addition, the factors showed acceptable levels of internal reliability and consistency (Cronbach's alpha = 0.0.65). Cronbach’s alpha coefficients were computed to determine the reliability of each factor. The data were analyzed by frequency, percentage, and chi-square since the variables in question included three (3) attributes for “yes”,  “sometimes”, and “no”. Demographic related characteristics including age and sex were analyzed using frequencies, percentages, means, and standard deviations. In addition, the independent samples t-test provided information about whether the distributions of scores in the two independent groups were drawn from two identical population distributions (Marsh et al. 1988). The data analysis used a t-test to identify differences attributed to the gender variable and used ANOVA to identify interval independent variables and the statistically significant differences between the countries.

## Results and Discussion

The first question of the study asked whether children’s nutrition and weight have been affected during the COVID-19 pandemic stay-at-home lockdown period in Saudi Arabia, the United Kingdom, and Turkey. The survey focused on the quantity and quality of children’s diets, as reported in Table 1.

**Table 1:** Children’s main meals per day

	Frequency	Percentage	Mean	Std. Deviation
1.00	12	3.6%	2.68	.680
2.00	104	31.5%		
3.00	197	59.7%		
4.00	12	3.6%		
5.00	5	1.5%		
Total	330	100%		

Table 1 reveals the responses that parents obtained from asking their children this question. It shows that most of the study population (59.7%) eats three main meals per day, while the percentage of those who eat only two main meals per day was 31.5%. The percentage of those who eat four main meals per day was 3.6%, and the percentage of those who eat only one main meal per day was 3.6%. Those who eat five main meals per day constituted 1.5% of the sample. The mean was 2.68, with an average score of 0.68.

On the other hand, Table 2 indicates that the percentage of the study population that ate two small meals per day was 38.8%, while the percentage of those who ate three small meals per day was 37.6%, and the percentage of those who eat only one small meal per day was 13.3%. The percentage of those who ate four small meals per day was 7.3%, and the percentage of those who ate five small meals per day was 1.5%. In addition, the percentage of those who ate no small meals during the day was 0.9%, while the percentage of those who ate six to ten small meals per day was 0.3%. The mean was 2.45 small meals per day, with an average score of (1.01).

**Table 2:** Children's number of small meals per day

	Frequency	Percentage	Mean	Std. Deviation
.00	3	.9	2.45	1.01
1.00	44	13.3		
2.00	128	38.8		
3.00	124	37.6		
4.00	24	7.3		
5.00	5	1.5		
6.00	1	.30		
10.00	1	.30		
Total	330	100.0		

The third question asked the parents about changes in their children’s eating habits during the COVID-19 lockdown, as per Table 3. Table 3 reveals that the averages for food is prepared at home; children drink enough water; children are eating fresh, unprocessed food; and children drink enough milk per day; were 2.94, 2.55, 2.51, and 2.35, respectively.

**Table 3:** The Nutrition system of Saudi, Turkish, and British children during the COVID-19 pandemic lockdown

		No	Sometimes	Yes	Mean	SD	Order
1. There are specific times for eating	f	77	86	167	2.27	0.82	6
	%	23.3	26.1	50.6			
2. The child is eating fresh, unprocessed food	f	29	103	198	2.51	0.65	3
	%	8.8	31.2	60.0			
3. The child eats more vegetables than before the lockdown	f	160	85	85	1.77	0.83	12
	%	48.5	25.8	25.8			
4. The child eats more fruits than before the lockdown period	f	140	77	113	1.92	0.87	9
	%	42.4	23.3	34.2			
5. The child drinks enough water	f	31	85	214	2.55	0.66	2
	%	9.4	25.8	64.8			
6. The child eats chocolate or sweets more than before the lockdown	f	135	92	103	1.90	0.84	10
	%	40.9	27.9	31.2			
7. Unhealthy food increased during the lockdown	f	208	51	71	1.58	0.82	16
	%	63.0	15.5	21.5			
8. The child eats enough meat	f	72	88	170	2.30	0.80	5
	%	21.8	26.7	51.5			
9. Child eats enough fish	f	191	80	59	1.60	0.77	15
	%	57.9	24.2	17.9			
10. The child eats more small meals than before the lockdown	f	111	83	136	2.08	0.86	7
	%	33.6	25.2	41.2			
11. Child eats more cakes, biscuits, and cupcakes than before the lockdown	f	136	77	117	1.94	0.88	8
	%	41.2	23.3	35.5			
12. The child will eat frozen foods such as pizza, nuggets, pies… more than before the lockdown period	f	214	61	55	1.52	0.76	17
	%	64.8	18.5	16.7			
13. The child drinks sweetened juices more than before the lockdown period	f	188	70	72	1.65	0.82	13
	%	57.0	21.2	21.8			
14. The child drinks enough milk per day	f	65	83	182	2.35	0.79	4
	%	19.7	25.2	55.2			
15. The child drinks more milk than prior the lockdown	f	191	71	68	1.63	0.80	14
	%	57.9	21.5	20.6			
16. The child drinks soft drinks	f	241	52	37	1.38	0.68	18
	%	73.0	15.8	11.2			
17. Food is prepared at home	f	6	7	317	2.94	0.30	1
	%	1.8	2.1	96.1			
18. The child's weight increased during the lockdown	f	167	54	109	1.82	0.90	11
	%	50.6	16.4	33.0			
Mean					2.21		

Although the parents’ attention to children’s nutrition was very high, there were also positive answers about unhealthy nutrition ingredients. For example, they indicated that children were drinking more soft drinks, sweetened juices, juice blends and fruit juice; and eating more frozen food such as pizza, nuggets, and pies; and chocolate, sweets, cakes, biscuits, and cupcakes than before the lockdown.

However, parents' responses showed that a high percentage of children were eating meat, but a lower percentage of them were eating fish. The responses also revealed a good percentage of children drinking milk. It was very important to observe the main meal timing for the family and children. Responses indicated that 50.6% of families followed a fixed schedule for meals during COVID-19 lockdown. This is important and is considered one of the basics in teaching children to maintain well-organized meals, including mealtimes.

In addition, when parents were asked whether the consumption of unhealthy food increased during the lockdown, 63% responded “no.” However, when the parents were asked whether the child's weight increased during the lockdown), 50.6% responded “no,” which means that increases in children’s weight were not due to unhealthy food and may be attributed to other factors such as the limited activities that they can engage in at home, the long hours of using digital games, and the unstable system of sleeping (children tend to play more and sleep less at inconsistent times). Finally, the parents' responses to item 11 also revealed a convergence in the responses of the parents on whether cake, biscuits, and cupcakes are unhealthy or healthy food: 41.2% responded “no” while 35.5% responded “yes.”

In addition, 50.5% of the responses of the Saudi participants to the direct question about the child’s weight increase during the lockdown period were “no.” This high rate raises a concern regarding the causes of weight increase among Saudi children, despite the fact that food was home-prepared, and quick meal-ordering was limited. The Turkish response in this regard was 44.3% “no,” and the British response was 58.1%. To the question of “Unhealthy food increased during the lockdown,” 72.9% of Turkish participants and 61.3% of the British participants answered “no.”

The second question is whether there are any statistically significant differences in child nutrition during the COVID-19 pandemic lockdown in Saudi Arabia, Turkey, and the United Kingdom, attributable to nationality. To determine the effect of the child’s nutritional system during this period, an ANOVA analysis was conducted. The results suggested significant differences, as shown in Table 4.

**Table 4:** ANOVA analysis of the effect of child’s nutritional system and their nationality

	Sum of Squares	Df	Mean Square	F	Sig
Between Countries	492.523	2	246.261	10.481	.000
Within Countries	7683.432	327	23.497		
Total	8175.955	329			

Table 4 clearly illustrates that there are statistically significant differences in child nutrition during the COVID-19 pandemic lockdown that are attributed to nationality. Table 5 presents the Scheffe’s test results to show the significance of differences in the child’s nutrition during the COVID-19 lockdown period by nationality.

**Table 5:** The Scheffe's post-test of the significance of the differences in children's nutrition based on their nationalities

(I) Nationality	(J) Nationality	Mean difference (I − J)	Std. Error	Sig
Saudi	Turkish	− 3.01284-^*^	.67405	.000
	British	− 1.46399-	.70544	.118
Turkish	Saudi	3.01284^*^	.67405	.000
	British	1.54885	.84537	.188
British	Saudi	1.46399	.70544	.118
	Turkish	− 1.54885-	.84537	.188

It is clear from Table 5 that there were differences in the nutrition systems of Saudi children and Turkish children and the differences favored Turkish children. Moreover, the nutritional system of Turkish and British children was better than that of Saudi children during the COVID-19 pandemic lockdown.

The third question was whether there were any statistically significant differences in child nutrition attributed to gender during the COVID-19 pandemic stay-at-home period in Saudi Arabia, the United Kingdom, and Turkey. To answer this question, an independent samples test t-test was conducted. The results illustrate the significance of the differences in child nutrition during the COVID-19 pandemic lockdown that are attributed to gender. Table 6 shows the results. There are statistically significant differences in children’s healthy nutrition systems during the COVID-19 pandemic lockdown attributed to the gender variable. The differences were in favor of males; the nutritional system of boys was better than that of girls during the COVID-19 pandemic lockdown. However, research shows that boys weigh more than girls (Ratajczak & Petriczko, 2020) and girls as young as three years of age are already invested in having a thin body (Harriger et al. 2010).

**Table 6:** T-test results of children’s healthy nutrition based on their gender

Child’s gender	N	Mean	Std. Deviation	T	Sig. (2-tailed)
Male	160	39.1625	5.09147	− 2.169	0.03
Female	170	40.3471	4.82751		

## Conclusions and Recommendations

With the spread of COVID-19, social media and magazines in Saudi Arabia are discussing the lockdown and how people are gaining weight. Researchers anticipate that many children may experience higher-calorie diets during the pandemic response due to the COVID-19 lockdown (Rundle et al. 2020; Milano Cities Changing Diabetes Atlas 2020). This raises the question of this research: Has child nutrition and weight been affected during the COVID-19 pandemic stay-at-home (Lockdown) period among children?

Furthermore, the social distancing and stay-at-home orders issued in cities across the globe increased screen time, video games, and cooking at home (Wilde, 2020). Screen time is associated with being overweight in childhood, likely because of the dual issues of sedentary time and the association between screen time and snacking (Marsh et al. 2013). Moreover, hearing or reading continuously about COVID-19 from media can be stressful, thus leading to subjects overeating, especially “comfort foods” that are rich in sugar and high in fat (Yılmaz & Gökmen, 2020). In addition, during their early years, children heavily depend on their parents or on their caregivers and their eating behaviors are mostly affected by them (Naserpoor 2018). The results from this study showed that parents in the three studied countries were paying attention to their children’s nutrition during the lockdown and they were preparing food at home (96.1%) during the COVID-19 pandemic. This contradicts what was circulated on social media about the linkage between stay-at-home cooking and weight gain. The participants’ responses to the direct question of whether their children gained weight due to eating unhealthy meals were “no” for 63.0% of respondents, which is above average. This study raised considerations about the effect of social media on people’s beliefs and attitudes. Parents should think twice before listening to what is being said in social media to reduce its influence on them. On the other hand, people in social media during this ongoing pandemic should not exaggerate or influence other people’s beliefs or attitudes.

The participating parents in the present study also emphasized that their children were neither consuming sweetened or soft drinks, nor eating frozen foods such as frozen pizza during the COVID-19 lockdown. Parents also made certain that their children had healthful meals and were drinking appropriate quantities of water at home. Children imitate their parents and view them as role models not only in the type of meals they have, but also in the way they eat their food (Gatus, 2015; Rangelov et al. 2016; Sawicka et al. 2017). Therefore, parents have a huge influence on constructing their children’s eating behaviors and habits as they can encourage them to have healthful meals and stay home. However, one of the limitations of this research is that the parents may have exaggerated the measures taken by them to maintain healthy diets for their children, rather than accurately reporting them. This bears mentioning while discussing the results.

The fear of disease and death, as well as the restrictions of individual freedom, worsened the stress load and produced an alteration of habitual behaviors leading parents to take good care of their children and their nutrition. This contradicts the findings of Von Hippel and Workman (2016). The researchers found that increases in student weight and the prevalence of obesity across three school years occur only during summer recess. This may also be considered by researchers for further longitudinal studies to understand this connection.

Another important factor in children’s nutritional systems is the number of small meals offered to children between main meals. Offering children three small meals or more helps to keep them healthy during the lockdown period, alongside playing video games, or using technology in their studies. Furthermore, frequent family meals are associated with positive family bonding, and it shows parents as positive role models for their children regarding food habits, meal types, and eating styles (Welsh et al. 2011; Martin‐Biggers et al. 2017).

Due to the conditions of the COVID-19 pandemic, children had enough quantities of meat (51.5%); but fewer quantities of fish (17.9%). Parents need to know the basic principles of meal preparation for children and the portions of different types of proteins children require in their meals (Gondek et al. 2018). Furthermore, most children consumed fresh food (60.0%). This highlights the role of parents in offering healthy balanced meals, which is consistent with the conclusions of Vittrup and McClure (2018). As displayed in Table 3, children in the three studied countries showed limited consumption of vegetables and fruits among the scored percentages that were convergent. This is also consistent with the findings of Pate et al. (2015), who concluded that most children between the ages of two and five consume an insufficient amount of fruit or vegetables to meet the dietary recommendations of Americans. Furthermore, researchers have argued that the nutritional behaviors that children acquire at an early stage when they do not consume sufficient amounts of fruit or vegetables will appear later in their adolescent behaviors, and increase potential health problems including obesity (Black, 2019; Leal et al. 2017).

Comparing the three studied countries and the behavior of parents or caregivers regarding their children’s nutritional systems revealed there were differences in the nutritional systems among the children of Turkey and Kingdom of Saudi Arabia in favor of Turkish children, as it is noted that there are specific timings for eating among Turkish children (68.6%), while Saudi children scored (44.4%), the weight gain of Turkish children was (32.9%), while Saudi children scored (33.3%), which is convergent. Moreover, 72.9% of the Turkish participants responded “no” to the question of whether there had been an increase in unhealthy food consumption during the lockdown period, versus 60.1% of the Saudi participants.” This confirms that Turkish families value the importance of healthy meals and confirms the findings of Ayranci et al. (2009).

Healthy food habits and behaviors include drinking water, eating vegetables and fruits, and for some individuals drinking milk might also be considered a healthy food habit. Turkish children scored higher than Saudi and British children. This calls for further research to examine Turkish nutritional habits, which could educate Saudi and British communities about young children’s healthy nutritional systems.

When parents in the three studied countries were asked whether their children were having cookies, cupcakes, or biscuits, 36.9% of parents in Saudi Arabia responded “yes.” The other two countries were convergent and parents responded with “no” (36.4%, 0.86). This raises questions about whether Saudi Arabian parents consider this type of food to be healthy.

This study also concluded that there were statistically significant differences in favor of boys regarding eating healthy foods, and this invites parents to focus on their daughters' nutrition as they grow rapidly during this important stage in their lives (Schanzenbach & Thorn, 2020). It supports what researchers concluded about the importance of communicating with families regarding their children’s nutrition (Tovar et al. 2015). Young children learn food habits and behaviors through the environment in which they grow up and imitate their friends and peers. This highlights the importance of paying attention to children's friends and their strong influence in eliminating unwanted habits (Szczepańska et al. 2015). Therefore, it is recommended that parents focus on their daughters’ eating habits and behaviors to help them have better nutritional habits.

In summary, during the COVID-19 pandemic, one of the clearest ways in which social media harmed families and children is through the spread of misinformation. These findings are valuable as they highlight the importance of recognizing and understanding the role of parents in their children’s nutrition, particularly during the ongoing pandemic. This is a comparative study that compared children’s nutrition during the lockdown period for three countries: Saudi Arabia, Turkey, and the United Kingdom. It showed that there is great attention on the part of the parents to providing healthy home-cooked meals for their children, and offering them enough healthy food, and water. It is an important lesson for parents to focus on their children’s nutrition during the lockdown period, with a particular focus on girls’ nutrition.

### Recommendations

Turkish children eat healthy food, and more research needs to be done on the eating habits of Turkish families. This research did not study the influence of parental nutrition knowledge or healthy-eating attitudes on children’s nutrition and health. These should be a focus for future research.

In addition, public health, social services, and media, especially social media, can support healthy eating by requiring innovative approaches to addressing food insecurity within the constraints of social distancing or full stay-at-home orders. Moreover, as UNICEF concentrates research efforts on children in poor countries, more research should be conducted on the norms of children in rich and middle-income countries.

It is vital to conduct further research regarding the amounts of foods, like cakes and biscuits, offered to children, as these were foods not considered a cause of weight gain by many parents in Saudi Arabia. Moreover, it is important to conduct more research on physical activities for children during the pandemic. Lastly, this research faced difficulty in reaching samples from other countries (UK- Turkish).
